# Supplementary material for: Does Aggressive Surgery Mean Worse Quality of Life and Functional Capacity in Retroperitoneal Sarcoma Patients?—A Retrospective Study of 161 Patients from China
Source: Cancers (Basel). 2022 Oct 19;14(20):5126. doi: 10.3390/cancers14205126 (PMC9600768; doi:10.3390/cancers14205126)
Supplement: Supplementary file 1 [file cancers-14-05126-s001.zip › cancers-1950393-supplementary.pdf]

Table S1. Baseline characteristics for patients who died at last follow-up.

Characteristics	Total (*n* = 99)
Gender	
Male	47 (47.5)
Female	52 (52.5)
Age, years mean (SD)	56.2 (13.7)
ASA score	
1	66 (66.7)
>1	33 (33.3)
Symptoms	
Yes	59 (59.6)
No	40 (40.4)
Tumor burden, cm mean (SD)	19.72 (10.1)
Histologic subtypes	
WDLPS	26 (26.3)
DDLPS	34 (34.3)
LMS	22 (22.2)
SFT	3 (3.0)
Others	14 (14.2)
FNCLCC	
Grade 1	21 (21.2)
Grade 2	31 (31.3)
Grade 3	47 (47.5)
Location	
Left	48 (48.5)
Right	51 (51.5)
Multifocality	
Yes	11 (11.1)
No	88 (88.9)
Radiation	
Yes	5 (5.1)
No	94 (94.9)
Chemotherapy	
Yes	16 (16.2)
No	83 (83.8)
MVR	
Yes	60 (60.0)
No	39 (40.0)
Operative time, hours mean (SD)	4.1 (1.6)
Estimated blood loss, ml median,IQR	600 (300-1500)
Packed RBC transfusion	
Yes	52 (52.5)
No	47 (47.5)
ICU Stay	
Yes	48 (48.5)
No	51 (51.5)

Severe postoperative adverse events	
Yes	23 (23.2)
No	76 (76.8)
Postoperative Hospital Stay, days mean (SD)	23.5 (17.3)
Disease recurrence	
Yes	77 (77.8)
No	22 (22.2)

Table S2. Baseline characteristics for responders and non-responders.

Characteristics	Responders（*n* = 161）	Non-responders (*n* = 59)	*p*
Gender			0.013
Male	76 (47.2)	39 (66.1)	
Female	85 (52.8)	20 (33.9)	
Age, years mean (SD)	55.2 (13.2)	58.3 (11.9)	0.117
ASA score			0.119
1	105 (65.2)	45 (76.3)	
>1	56 (34.8)	14 (23.7)	
Symptoms			0.031
Yes	51 (31.7)	28 (47.5)	
No	110 (68.3)	31 (52.5)	
Tumor burden, cm mean (SD)	16.3 (9.2)	16.9 (9.6)	0.699
Histologic subtypes			0.511
WDLPS	72 (44.7)	24 (40.7)	
DDLPS	32 (19.9)	14 (23.7)	
LMS	23 (14.3)	7 (11.9)	
SFT	18 (11.2)	4 (6.8)	
Others	16 (9.9)	10 (16.9)	
FNCLCC			0.643
Grade 1	69 (42.9)	23 (39.0)	
Grade 2	51 (31.7)	22 (37.3)	
Grade 3	30 (18.6)	14 (23.7)	
Unknow	11 (6.8)	0 (0.0)	
Location			0.752
Left	83 (51.6)	29 (49.2)	
Right	78 (48.4)	30 (50.8)	
Multifocality			0.412
Yes	11 (6.8)	6 (10.2)	
No	150 (93.2)	53 (89.8)	
Radiation			0.601
Yes	16 (9.9)	4 (6.8)	
No	145 (90.1)	55 (93.2)	
Chemotherapy			0.110
Yes	17 (10.6)	2 (3.4)	
No	144 (89.4)	57 (96.6)	

MVR			0.621
Yes	77 (47.8)	26 (44.1)	
No	84 (52.2)	33 (55.9)	
Operative time, hours mean (SD)	3.5 (1.6)	3.5 (13.4)	0.811
Estimated blood loss, ml median,IQR	567.8 (725.8)	530.2 (866.0)	0.747
Packed RBC transfusion			0.591
Yes	38 (23.6)	16 (27.1)	
No	123 (76.4)	43 (72.9)	
ICU Stay			0.251
Yes	85 (52.8)	26 (44.1)	
No	76 (47.2)	33 (55.9)	
Severe postoperative adverse events			0.437
Yes	9 (5.6)	5 (8.5)	
No	152 (94.4)	54 (91.5)	
Postoperative Hospital Stay, days mean (SD)	16.2 (10.4)	15.8 (9.2)	0.811
Disease recurrence			0.207
Yes	31 (19.3)	16 (27.1)	
No	130 (80.7)	43 (72.9)	
